# The Effect of Sulphate Anions on the Ultrafine Titania Nucleation

**DOI:** 10.1186/s11671-017-2144-3

**Published:** 2017-05-23

**Authors:** Volodymyr O. Kotsyubynsky, Ivan F. Myronyuk, Volodymyr L. Chelyadyn, Andriy B. Hrubiak, Volodymyr V. Moklyak, Sofia V. Fedorchenko

**Affiliations:** 1 Vasyl Stefanyk Precarpathian National University, 57 Shevchenko str, Ivano-Frankivsk, 76018 Ukraine; 2 Institute of Materials Science I.M. Frantsevich, 3 Academic Krzhizhanovskii Str, Kyiv, 03680 Ukraine; 3 Institute of Metal Physics, National Academy of Science, 36 Acad. Vernadsky Boulevard, Kyiv, 03680 Ukraine

**Keywords:** Hydrolysis, Polycondensation, Nucleation, Anatase, Sulphate anions

## Abstract

The phenomenological model of sulphate anions effect on the nanodispersed titania synthesis during hydrolysis of titanium tetrachloride was studied. It was proposed that both chelating and bridging bidentate complexes formation between sulphate anions and octahedrally coordinated [Ti(OH)_h_(OH_2_)_6−h_]^(4−h)+^ mononers is the determinative factor for anatase phase nucleation.

## Background

Ultrafine TiO_2_ has wide range of highly promising applications in many different fields—from the environmental oriented photocatalytic system, such as degradation of hazardous organic compounds [1], waste water cleaning [2], direct decomposition of NO_*x*_, SO_*x*_ and air purification [3] to novel fields of industry—sensor materials [4] and solar cells [5]. Phase composition, particle size and surface state are the most important characteristics that determine catalytic reactivity, photosensitivity and adsorption properties of TiO_2_. For example, the decrease of particle size of titania leads to the rapid increase of catalytic activity [6]. At the same time, the photocatalytic properties of titania are very sensitive to phase composition of TiO_2_ polymorphs such as anatase, brookite and rutile [7].

Choice of titania synthesis method with the control of its physical and chemical parameters is crucially important determinants of resulted compositions. The preparation of nanosized TiO_2_ is possible by sol-gel [8], chemical precipitation [9], microemulsion [8] and hydrothermal [10] methods. The sol-gel method is the most flexible technique for nanosized oxide preparation. The variations of primary precursor types, hydrolysis conditions, temperature and pH of the reaction medium allow the control of nanoparticle nucleation and growth. The sol-gel method of titania obtained is typically based on the reactions of titanium alkoxides Ti(OR)_n_ hydrolysis. The change of these expensive chemicals to the cheaper precursor such as TiCl_4_ is very promising for a large scale manufacture of nanosized TiO_2_. A promising advantage of TiCl_4_ application is the possibility of polycondensation reactions controlled by additive ions with the use of the predicted nucleation of the specified phase of titania.

The aim of this paper was to investigate the effects of SO_4_^2−^ anions on the oligomer polycondensation and oxide network formation during titania nucleation for the sol-gel process based on TiCl_4_ hydrolysis.

## Methods

Titanium tetrachloride TiCl_4_ (Merck, 99.9%; specific density 1.73 g/cm^3^ at 20 °C) was cooled to 0 °C and hydrochloric acid (36.0% aqueous solution) was added with further hydrogen chloride evaporation. The TiCl_4_ to hydrochloric acid ratio was 2:1. Aqueous solution of sodium hydrocarbonate was added dropwise to sol of titanium oxychloride TiOCl_2_ to get pH of 5.0–5.5 under vigorous stirring. Gel formation was observed during all pH increasing process. The suspension of nanoparticles was kept at 80 °C for 3 h with the further washed with distilled water to remove Na^+^ and Cl^−^ ions. Precipitated TiO_2_ was dried at the 150 °C, and obtained xerogel was marked as S1. The S2 material synthesis process was carried out likewise, but crystalline-dried Na_2_SO_4_ was added directly to titanium tetrachloride on the stage of TiCl_4_ hydrolysis.

Diffraction patterns were obtained with the diffractometer DRON-4-07 equipped with an X-ray tube BSV28 (Cu K_α_ radiation, 40 kV, 30 mA), a Bragg-Brentano geometry-type and a Ni K_β_-filter. A qualitative analysis was carried out with the use of ICSD structural models. The structural models for anatase and rutile were based on the ICSD #92363 and ICSD #24780, respectively. Copper powder annealed in vacuum (850–900 °C for 4 h) with an average grain size of about 50 μm was used as a reference sample to determinate instrumental peak broadening. Full width at half maximum (FWHM) for a diffraction peak of this reference sample at the 2θ = 43.38° was 0.129°; therefore, it made it possible to distinguish anatase and brookite phases. The size of the coherently scattering domains was calculated by the Scherrer equation: $D=\frac{K\lambda}{\beta cos\theta}$, with *K* is the Scherrer constant (*K* = 0.9), *λ* is the wavelength (0.154 nm), *β* the FWHM (in radians), and *θ* is the peak angular position. We used the combination of Gauss and Cauchy (dominated) functions as a profile shape.

Infrared spectra were recorded with Thermo-Nicolet Nexus 670 FTIR spectrometer in the 4000–400 cm^−1^ region. The TiO_2_/KBr mixture after vibrating milling was pressed into pellets and measured in the transmission mode.

The morphology of sample powders was studied by transmission electron microscopy (TEM) with a 100-kV microscope JEOL JEM-100CX II. The microscopic copper grid covered by a thin transparent carbon film was used as a specimen support for TEM researches.

## Results and Discussion

**Fig. 1:**
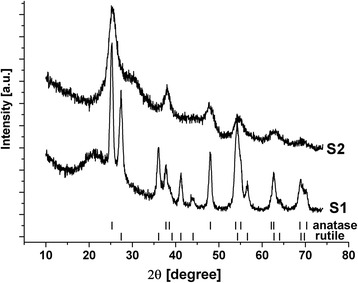
XRD patterns of S1 and S2 materials

The presence of sodium sulphate in the reaction medium significantly affected the phase composition of the obtained materials (Fig. 1). The material synthesized in the absence of Na_2_SO_4_ additive (S1) was a mixture of anatase and rutile with the relative phase contents of 65 ± 4 and 35 ± 5 wt %, respectively. The average size of the coherently scattering domains (CSD) was about 14 nm for anatase and 9 nm for rutile, so both phases are good crystallized. Meanwhile, the part of the material is close to amorphous state as the presence of the halo on XRD pattern for 2θ = 16–32^o^ is evident. According to synthesis conditions, the formation of non-titania phase is unlikely to occur. As a result, the material consists of separated regions with different crystallinity degrees. The specific surface area of S1 sample was about 152 m^2^ g^−1^. The material S2 was close to amorphous ultrafine titania with clear structural features of anatase. The halo on XRD pattern is also observed in this case but it is relatively narrowed and shifted to larger 2θ values. The average size of CSD was about 4–5 nm (the analysis is complicated by low crystallinity of the material). The specific surface area for S2 sample was increased to 328 m^2^ g^−1^.

**Fig. 2:**
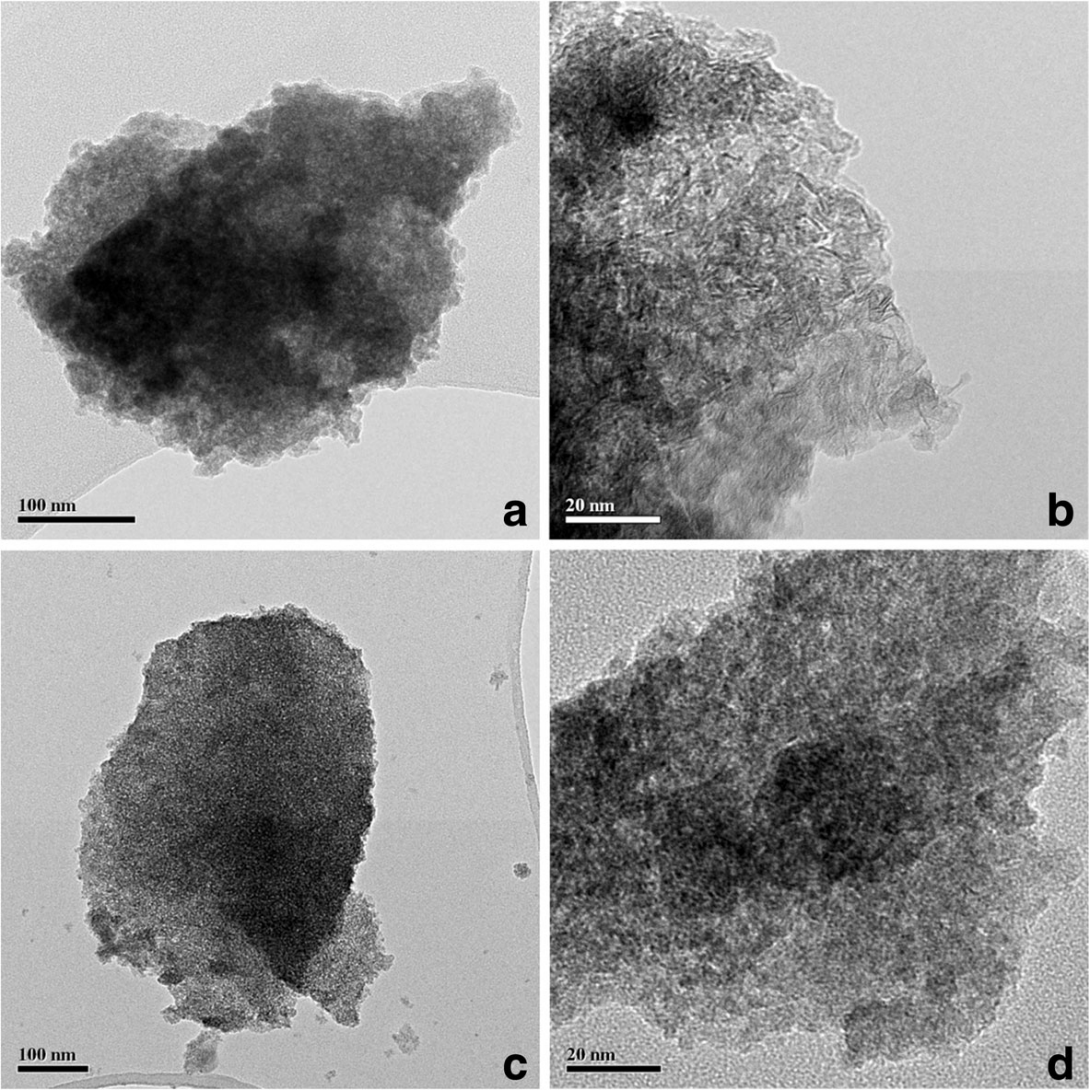
TEM images of the samples S1 (**a**, **b**) and S2 (**c**, **d**)

**Fig. 3:**
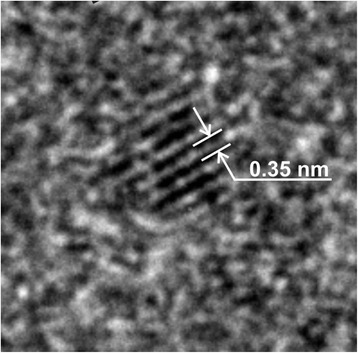
HR TEM images of S2 material with the fringes from {101} planes

TEM images of S1 sample (Fig. 2a) do not allow to make clear conclusions about its morphology but the observed agglomerates consisted of the lamellar-like primary particles with the sizes of 10–15 nm. Furthermore, there is no evidence of crystalline area boundaries (Fig. 2b). The S2 sample had bubble-like morphology of the agglomerates (Fig. 2c, d). HR TEM showed high crystallinity of some grains of this material (Fig. 3) with the interplanar distances of 0.34–0.37 nm. The obtained interplanar spacing corresponds to the (101) plane of anatase (0.352 nm). This indicates that the preferred growth direction of CSD (crystallites) is [010] crystallography axis. This result led to conclusions that the anatase nanocrystals with oxygenated surfaces have developed facets in the 〈010〉 direction [11].

**Fig. 4:**
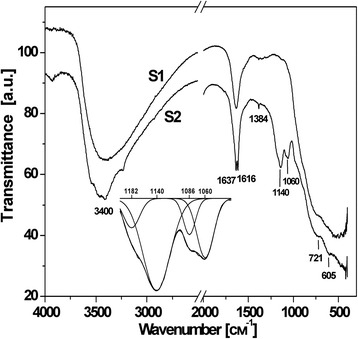
FTIR spectra of S1 and S2 materials

More information about synthesized materials was obtained by FTIR spectroscopy. The broad absorption area around 3400 cm^−1^ indicates the presence of chemisorbed OH-groups on the surface of titania particles (ν-OH modes) (Fig. 4) [12]. The shift of the ν-OH bands from typical 3700–3600 to about 3400 cm^−1^ can be caused by the presence of hydrogen bonding [13]. The band around 1600 cm^−1^ demonstrates the presence of molecularly adsorbed water (δ-H_2_O modes) [14]. The higher crystallinity degree for S1 sample causes the formation of relatively more distinct absorption bands in the titania characteristic area (400–700 cm^−1^) [15].

The additional absorbance band on the FTIR patterns for S2 materials at 1139 and 1060 cm^−1^ corresponds to chemisorbed SO_4_^2−^ ions [16]. A sharp low intensity band 1384 cm^−1^ is typical for the metal oxides modified by sulphate ion bands and assigned to S=O stretching frequency. Meanwhile, S=O–H coordination is unlikely because the absorption band will shift in low-frequency area up to 1325 cm^−1^ in this case. There are two different variants of SO_4_^2−^ immobilization on the titania surface—a chelating bidentate complex formation with coordination to one metal ion through two oxygens or bridged bidentate complex formation using bonding through two metal ions; both complexes belong to C2v point group. Bridged bidentate SO_4_^2−^ anions coordinated to Ti^4+^ have characteristic stretching frequencies in the 930–1200 cm^−1^ range, and major absorption peak at 1148 cm^−1^ is attributed to asymmetric stretching vibrations [17]. The bands in the 1300–900 cm^−1^ region were observed for SO_4_^2−^/TiO_2_ system, and the peaks at 1217, 1134, 1044, and 980 cm^−1^ were identified in [18] as characteristic frequencies of a bridge bidentate SO_4_^2−^ coordinated to metals. According to [19], bridged bidentate complex has four absorption bands at 1195–1160, 1110–1105, 1035–1030 and 990–960 cm^−1^, which are assigned to the asymmetric and symmetric stretching frequencies of the S=O and S–O bonds.

The conclusion about energetic favorability of chelating complex formation where SO_4_^2−^ anions are coordinated to Ti atoms through two oxygen was made on the base of sulphated titania investigation with the use of DFT calculation [20]. The formation of chelate sulphate complex corresponds to skeletal FTIR band at 1201 cm^−1^ [21] as chelating bidentate complex has four bands at 1240–1230, 1125–1090, 1035–995 and 960–940 cm^−1^ which are assigned to the asymmetric and symmetric stretching frequencies of S=O and S–O bands [19].

Deconvolution of the 1200–1000 cm^−1^ region of S2 material FTIR spectra revealed the presence of four bands at 1182, 1140, 1086 and 1060 cm^−1^. The absorption band at 1086 cm^−1^ is quite close to that of the chelating bidentate complex. Two bands of chelating and bridging bidentate complexes overlap each other so band at 1182 cm^−1^ can correspond to both types of complexes. The bands at 1060 and 1140 cm^−1^ imply that bridged bidentate complex is formed on the surface of the S2 sample.

We can suggest the following model of SO_4_^2−^ impact on titania nucleation at the stage of olation interaction between primary hydrocomplexes taking into account the results shown in [22]. The hydrolysis of TiCl_4_ leads to [Ti(OH_2_)_6_]^4+^ formation where Ti^4+^ ions are in the octahedral coordination with the next transformation to [Ti(OH)_h_(OH_2_)_6−h_]^(4−h)+^ monomers as a result of deprotonation. The hydrolysis ratio *h* is a function of pH and is determined by partial charge theory [23]. In these monomers, OH^−^ groups have thermodynamic advantages of the location in the octahedron equatorial planes, and H_2_O molecules primarily occupy the “vertex” position [24]. The products of hydrolysis are [Ti(OH)(OH_2_)_5_]^3+^ and [Ti(OH)_2_(OH_2_)_4_]^2+^ monomers when pH of reaction medium is close to 1. At pH = 3, the [Ti(OH)_2_(OH_2_)_4_]^2+^ and [Ti(OH)_3_(OH_2_)_3_]^+^ complexes coexist in solution. At pH = 4, the hydrolysis leads to the formation of the [Ti(OH)_3_(OH_2_)_3_]^+^ complexes, and in the range of pH = 6–8, the [Ti(OH)_4_(OH_2_)_2_]^0^ monomers are formed. The possibility of the titania polymorph formation is defined by the spatial organization of [Ti(OH)_h_(OH_2_)_6−h_]^(4−h)+^ primary monomers. [Ti(OH)_4_(OH_2_)_2_]^0^ monomers (in which OH groups occupy octahedron equatorial planes and H_2_O molecules are in the vertexes) form in neutral or alkaline mediums [20, 25]. Dimers are formed as a result of olation reaction between two primary monomers for which the octahedron coordination has a common edge outside the octahedron equatorial plane. After further polycondensation, the zigzag-like or spiral chain of [Ti_n_(OH)_4n_(OH_2_)_2_]^0^ polyhedrons are formed and the conditions for the anatase phase nucleation are created. The [Ti_mn_O_mn_(OH)_2mn_(OH_2_)_2m_]^0^ polymer is formed resulting from *m* linear structures of [Ti_n_(OH)_4n_(OH_2_)_2_]^0^ olation interaction. The nucleation of anatase phase is the result of octahedral merger by lateral planes of faces [26]. At the same time, the hydronium ions of the reaction medium interact with hydroxyl groups in the octahedron equatorial plane. If the hydronium ion concentration in the reaction medium increases, [Ti(OH)_h_(OH_2_)_6−h_]^(4−h)+^ monomers will form under *h* < 2 condition. Olation interaction between them leads to the polymer chain formation where monomers are linked by joint edges in octahedron equatorial planes, thus defining the precondition for rutile phase nucleation [25].

**Fig. 5:**
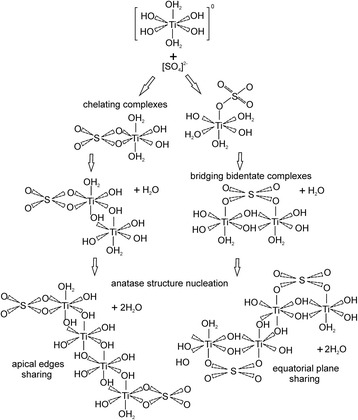
Proposed mechanisms of anatase phase nucleation with the formation of chelating and bridging bidentate SO_4_^2−^ complexes

The presence of SO_4_^2−^ ions in the reaction medium at pH about 5.5 will cause both Ti(SO_4_)(OH)_2_(H_2_O)_2_ chelating and Ti_2_(SO_4_)(OH)_6_(OH_2_)_2_ bridging bidentate complexes formation (Fig. 5). There are two different pathways of olation interaction between these complexes. Two monomers connect with each other or by sharing apical edges (chelating complexes influence) or in equatorial plane (bridging bidentate complexes influence) with the dehydrating of water molecule. At the next stage in both cases, the formation of skewed zigzag-like tetranuclear titanium complexes with the dehydrating of two water molecules takes place and the nucleation of anatase structure starts.

## Conclusions

The effect of SO_4_^2−^ anions on the titania nucleation during hydrolysis of titanium tetrachloride was studied. We concluded that the nucleation process is mainly controlled by pH of the reaction medium and SO_4_^2−^ anion presence. Sulphate anions form both chelating Ti(SO_4_)(OH)_2_(H_2_O)_2_ and bridging bidentate Ti_2_(SO_4_)(OH)_6_(OH_2_)_2_ complexes at the stage of titanium tetrachloride hydrolysis. We suggested the model with two pathways of olation interaction between titano-sulphate complexes when SO_4_^2-^ ligands stimulate screw polymer chains formation and the nucleation of TiO_2_ anatase phase.
